# Malaria Prevalence, Risk Factors and Spatial Distribution in a Hilly Forest Area of Bangladesh

**DOI:** 10.1371/journal.pone.0018908

**Published:** 2011-04-21

**Authors:** Ubydul Haque, Toshihiko Sunahara, Masahiro Hashizume, Timothy Shields, Taro Yamamoto, Rashidul Haque, Gregory E. Glass

**Affiliations:** 1 International Center for Diarrhoeal Disease Research Bangladesh, Mohakhali, Dhaka, Bangladesh; 2 Department of International Health, Institute of Tropical Medicine (NEKKEN) and the Global Center of Excellence Program, Nagasaki University, Nagasaki, Japan; 3 Department of Molecular Microbiology and Immunology, Johns Hopkins Bloomberg School of Public Health, Baltimore, Maryland, United States of America; University of Barcelona, Spain

## Abstract

**Background:**

Malaria is a major public health concern in Bangladesh and it is highly endemic in the Chittagong Hill Tracts where prevalence was 11.7% in 2007. One sub-district, Rajasthali, had a prevalence of 36%. Several interventions were introduced in early 2007 to control malaria. This study was undertaken to evaluate the impacts of these intensive early stage interventions on malaria in Bangladesh. This prevalence study assesses whether or not high malaria prevalence remains, and if so, which areas and individuals remain at high risk of infection.

**Methods and Principal Findings:**

A 2-stage cluster sampling technique was used to sample 1,400 of 5,322 (26.3%) households in Rajasthali, and screened using a rapid diagnostic test (Falci-vax). Overall malaria prevalence was 11.5%. The proportions of *Plasmodium falciparum*, *Plasmodium vivax* and infection with both species were 93.2%, 1.9% and 5.0%, respectively. Univariate, multivariate logistic regression, and spatial cluster analyses were performed separately. Sex, age, number of bed nets, forest cover, altitude and household density were potential risk factors. A statistically significant malaria cluster was identified. Significant differences among risk factors were observed between cluster and non-cluster areas.

**Conclusion and Significance:**

Malaria has significantly decreased within 2 years after onset of intervention program. Both aspects of the physical and social environment, as well as demographic characteristics are associated with spatial heterogeneity of risk. The ability to identify and locate these areas provides a strategy for targeting interventions during initial stages of intervention programs. However, in high risk clusters of transmission, even extensive coverage by current programs leaves transmission ongoing at reduced levels. This indicates the need for continued development of new strategies for identification and treatment as well as improved understanding of the patterns and determinants of parasitaemia.

## Introduction

Malaria is a major health burden that is widespread throughout tropical and subtropical regions of the world. In 2009, there were an estimated 225 million cases of malaria worldwide that accounted for approximately 781,000 deaths [1].

As in many at-risk countries malaria is a major public health concern in Bangladesh, where it is limited to 13 of the 64 administrative districts [2]. Malaria is largely seasonal in Bangladesh, with the major incidence occurring during the rainy season from April to October [3]. In 2009; 63,873 malaria cases and 47 confirmed deaths were reported from the 13 endemic districts [4]. Among these malaria-endemic districts, three located in the southeastern Chittagong Hill Tracts (CHT) region had a high burden that accounted for 90% of the total malaria morbidity and mortality in Bangladesh. Active case detection with rapid diagnosis tests (RDT) conducted in 2007 revealed that the total malaria prevalence in the CHT was 11.7% with a majority of infections being caused by *Plasmodium falciparum* (>90%) [5].

In 2006, Bangladesh was supported with $36.9 million USD from the Global Fund to support national malaria control program. As a result, a number of interventions have been introduced, including early diagnosis and treatment, distribution of long-lasting insecticide treated bed nets (LLINs) among 80% of households in endemic districts, re-treatment of insecticide-treated nets (ITNs) in 40% of households, enhanced vector surveillance and control, and an updated record keeping system. In 2008, a total of 568,661 nets were re-treated and 1,199,987 LLINs were distributed [4]. This project is now at the mid-stage and will continue until the end of 2012. A study to evaluate the effects of malaria control in Rajasthali, a sub-district with the highest prevalence (36%) in the 2007 survey, indicated that LLIN distribution (>90%) and the re-treatment of ITN (>40%) exceeded target goals by 2009, although the treatment seeking strategies by local peoples remained challenging: approximately a half of the population used drug venders for treatment of malaria-associated fever [6].

This study was undertaken to identify the environmental and socio-economic risk factors and their geographical patterns. The goal was to help us design cost-effective strategies for further malaria control.

## Results

### Characteristics of the study population

In Rajasthali 1,400 individuals were screened using the RDT. Out of 1400 samples, 161 (11.5%) were positive for either *P. falciparum* (158, 11.3%) or *P. vivax* (11, 0.8%). Co-infection was found in 8 samples (0.6%), which was more common than expected under an assumption of independent and homogeneous infection rate of the 2 species (1.24, 0.09%; Fisher's exact test, p<0.01). Individual and household characteristics are shown in Table 1. Bed nets (≥2) were used sparingly (43.6% of households), 80.8% of people regularly slept under bed nets, and 47.1% of people treated their bed nets with insecticide.

**Table 1:** Frequencies and odds ratios for potential risk factors for malaria infections (Rapid Diagnostic Test positives) in Rajasthali.

			Univariate (Unadjusted)			Multivariate (Adjusted)		
Variables	Total population	No. of malaria positives	OR	95% CI	P-Value	OR	95% CI	P-Value
**Sex**								
Female	832	70	1			1		
Male	568	91	2.08	1.49–2.89	0.001	1.66	1.16–2.39	0.006
**Age (years)**								
0–14	363	73	1			1		
>14–49	824	71	0.37	0.26–0.53	0.001	0.43	0.29–0.62	0.001
>49	213	17	0.34	0.20–0.60	0.001	0.33	0.18–0.61	0.001
**Tribe**								
Bengali	357	23	1			1		
Marma	593	53	1.43	0.86–2.37	0.172	1.14	0.61–2.12	0.682
Tripura	143	33	4.36	2.45–7.74	0.001	1.29	0.56–2.97	0.549
Tonchonga	230	32	2.35	1.34–4.12	0.003	1.34	0.67–2.68	0.409
Khiang & Chakma	77	20	5.10	2.63–9.88	0.001	1.54	0.60–3.92	0.369
**Education (years)**								
0	671	66	1			1		
1–5	312	42	1.43	0.94–2.15	0.092	1.34	0.86–2.07	0.194
6–10	366	51	1.48	1.0–2.19	0.047	1.23	0.81–1.87	0.329
>10	51	2	0.37	0.09–1.57	0.180	0.36	0.08–1.57	0.174
**Occupation**								
Service/Business	169	17	1					
Small business	118	19	1.72	0.85–3.46	0.131			
Day labor	304	42	1.43	0.79–2.61	0.238			
Agriculture	745	74	0.99	0.57–1.72	0.961			
Unemployed	64	9	1.46	0.62–3.47	0.388			
**Number of bed nets**								
≤2	789	108	1			1		
>2	611	53	0.60	0.42–0.85	0.004	0.65	0.45–0.95	0.028
**Treated bed net or LLIN ownership**								
No	68	9	1					
Yes	1332	152	0.84	0.41–1.74	0.646			
**All family members sleep under bed net**								
No	268	40	1					
Yes	1132	121	0.68	0.46–1.00	0.052			
**Forest**								
1^st^ Tertile	467	23	1			1		
2^nd^ Tertile	467	48	2.21	1.32–3.69	0.003	2.54	1.40–4.60	0.002
3^rd^ Tertile	466	90	4.62	2.86–7.45	0.001	7.40	2.72–20.12	0.001
**Altitude (**meter**)**								
≤50	883	78	1			1		
51–100	446	59	1.57	1.10–2.25	0.013	1.47	0.87–2.46	0.147
>100	71	24	5.27	3.06–9.08	0.001	3.30	1.50–7.28	0.003
**Type of floor**								
Mud	661	55	1			1		
Cement	112	9	0.96	0.46–2.00	0.919	1.29	0.59–2.83	0.517
Wood	627	97	2.02	1.42–2.86	0.001	1.25	0.74–2.10	0.402
**Household density**								
1–200	375	72	1			1		
201–500	327	32	0.46	0.29–0.71	0.001	1.68	0.86–3.28	0.126
501–1000	127	11	0.40	0.20–0.78	0.007	3.44	1.19–9.95	0.023
>1000	571	46	0.37	0.25–0.55	0.001	4.07	1.42–11.64	0.009
**Use of malaria control program**								
No	369	31	1			1		
Yes	1031	130	1.57	1.04–2.37	0.031	1.33	0.82–2.15	0.246

### Risk factor analysis

Risk factors were identified using univariate logistic regression analysis (Table 1). Sex, age, ethnicity, education, number of bed nets, extent of forest cover, altitude, floor construction materials, household density and treatment seeking preference to malaria control program were significant risk factors in univariate analysis. In univariate analysis, all family members sleeping regularly under bed net was marginally significant (P = 0.052).

In the multivariate analysis, a significant association with malaria was observed for the effects of sex, age group, having 2 or fewer bed nets, increased forest cover, elevation and household density was similar to the results observed for the univariate models. The risk of malaria infection in males was higher than in females (OR: 1.66, 95% CI 1.16–2.39). Children 0–14 years of age were more vulnerable than individuals 15–49 and ≥50 years of age. Possession of 2 or more bed nets was protective against infection. Increased forest coverage within 2 km from the household also proved to be a significant risk factor, and people living at higher altitudes were more vulnerable to infection. Based on univariate analysis, lower household density (1–200) was a risk factor for infection, while higher density (>1000) associated with a decreased risk. The effect of household density was, however, reversed in the multivariate model, where higher density (>1000) produced an increased risk.

### Geographic cluster

**Figure 1:**
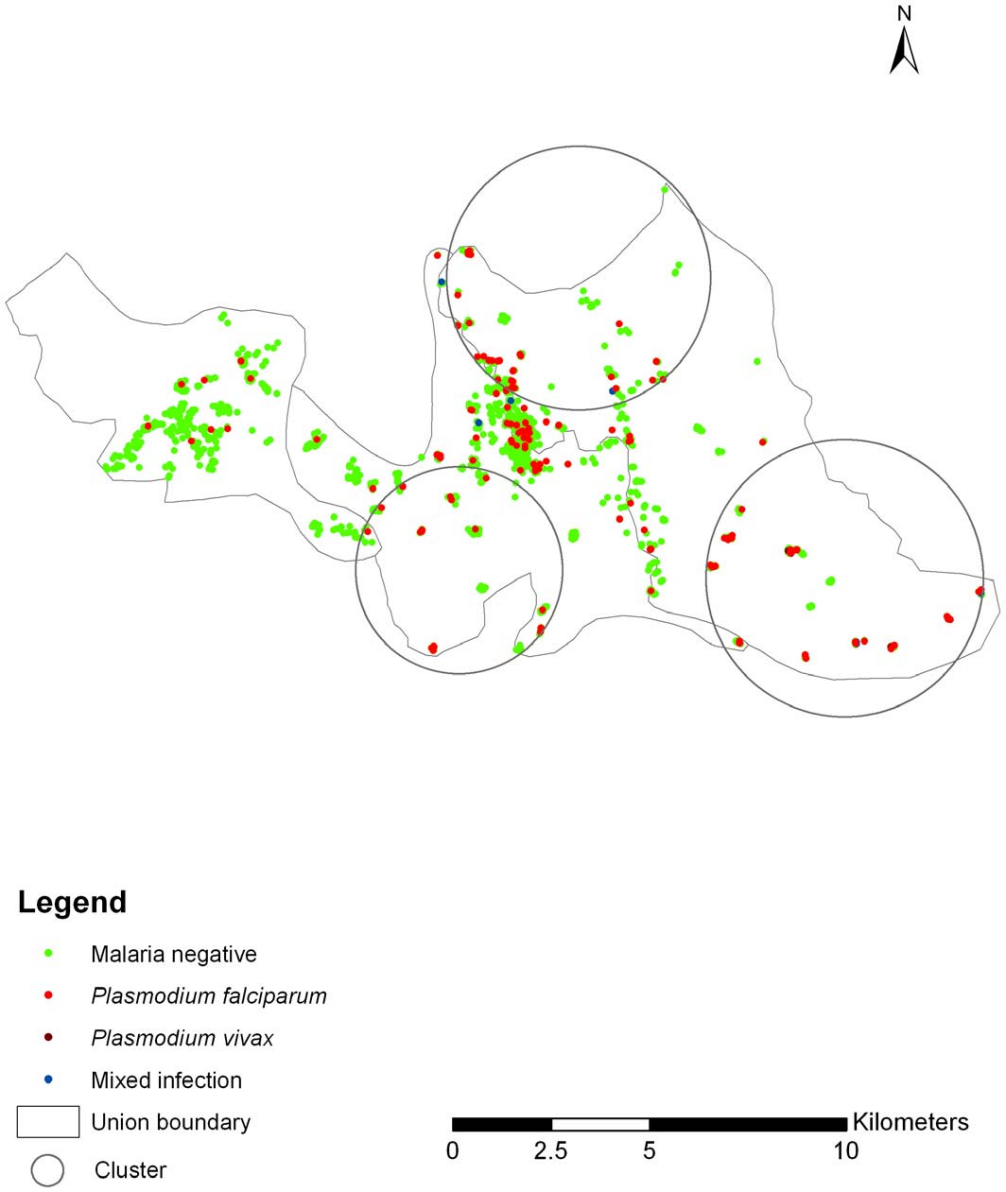
Spatial distribution of malaria prevalence in Rajasthali.

Identified spatial clusters of malaria risk are shown in Figure 1. Three statistically significant malaria clusters (Table 2) were observed in the study area and the most likely cluster was a geographically large area (8.12 km) in southeastern Rajasthali, with an relative risk of 3.39 (p = 0.001). We then compared characteristics of individuals in the three clusters (n = 852) with characteristics of the population outside of the clusters (n = 548) (Table 2). According to univariate analysis, ethnicity, occupation, treated bed net or LLIN ownership, forest cover, altitude, floor construction materials, household density and preference of treatment from the malaria control program proved significant (Table 3).

**Table 2:** Geographic cluster of malaria prevalence in Rajasthali.

Cluster	Population	No. of cases	Expected cases	Relative Risk	Log Likelihood Ratio	P-Value
**Most likely cluster**	136	43	15.64	3.39	22.59	0.001
**Secondary cluster**	219	44	20.44	2.84	15.02	0.001
	497	54	35.19	2.98	510.59	0.026
	5	3	0.18	19.17	6.86	0.182

**Table 3:** Comparison of potential risk factors between residents living within high malarial risk clusters and non-cluster areas.

			Univariate (Unadjusted)			Multivariate (adjusted)		
	Cluster	Non-cluster	OR	95% CI	P-Value	OR	95% CI	P-Value
Variables	Frequency	Frequency						
**Sex**								
Female	498	334	1					
Male	354	214	1.11	0.89–1.38	0.353			
**Age (years)**								
0–14	220	143	1					
>14–49	499	325	1.00	0.78–1.28	0.988			
>49	133	80	1.08	0.76–1.53	0.662			
**Tribe**								
Bengali	152	205	1			1		
Marma	356	237	2.03	1.56–2.64	0.001	1.13	0.77–1.66	0.528
Tripura	116	27	5.79	3.63–9.26	0.001	0.20	0.09–0.44	0.001
Tonchonga	153	77	2.68	1.90–3.78	0.001	0.53	0.33–0.84	0.007
Khiang & Chakma	75	2	50.58	12.23–209.21	0.001	3.85	0.71–20.92	0.118
**Education (years)**								
0	400	271	1					
1–5	190	122	1.06	0.80–1.39	0.702			
6–10	234	132	1.20	0.92–1.56	0.173			
>10	28	23	0 .82	0.47–1.46	0.510			
**Occupation**								
Service/Business	91	78	1			1		
Small business	78	40	1.67	1.03–2.72	0.039	0.99	0.55–1.82	0.988
Day labor	186	118	1.35	0.92–1.98	0.121	0.93	0.57–1.50	0.756
Agriculture	448	297	1.29	0.92–1.81	0.134	1.41	0.92–2.15	0.113
Unemployed	49	15	2.80	1.46–5.38	0.002	1.89	0.85–4.21	0.118
**Number of bed net**								
<2	487	302	1					
≥2	365	246	0.92	0.74–1.14	0.450			
**Treated bed net or LLIN ownership**								
No	31	37	1			1		
Yes	821	511	1.92	1.17–3.13	0.009	1.82	0.93–3.55	0.081
**All family members sleep under bed net**								
No	170	98	1					
Yes	682	450	0.87	0.66–1.15	0.337			
**Forest**								
1^st^ Tertile	143	324	1			1		
2^nd^ Tertile	298	169	4.00	3.04–5.25	0.001	3.42	2.40–4.88	0.001
3^rd^ Tertile	411	55	16.93	12.01–23.87	0.001	17.28	7.97–36.75	0.001
**Altitude (meter)**								
≤50	496	387	1			1		
51–100	289	157	1.44	1.13–1.82	0.003	1.13	0.74–1.71	0.574
>100	67	4	13.07	4.72–36.15	0.001	3.44	1.06–11.12	0.039
**Floor**								
Mud	303	358	1			1		
Cement	62	50	1.47	0.98–2.19	0.063	1.43	0.89–2.29	0.134
Wood	487	140	4.11	3.23–5.24	0.001	1.98	1.32–2.96	0.001
**Household density**								
1–200	336	39	1			1		
201–500	184	143	0.15	0.10–0.22	0.001	0.22	0.11–0.45	0.001
501–1000	52	75	0 .08	0.05–0.13	0.001	0.46	0.18–1.15	0.097
>1000	280	291	0 .11	0.08–0.16	0.001	0.64	0.26–1.60	0.342
**Malaria control program**								
No	123	246	1			1		
Yes	729	302	4.83	3.74–6.23	0.001	6.82	4.71–9.89	0.001

For the multivariate model, a significant association was observed between malaria positives and ethnicity, forest cover, altitude, floor construction, household density and treatment preference from the malaria control program. Among the tribes, most of Tonchonga, and Tripura communities lived within the malaria clusters. There was also relatively denser forest (2^nd^ and 3^rd^ tertiles) within the clusters. Clusters were associated with higher elevations (>100 meter; OR: 3.44, 95% CI 1.06–11.12). Within the clusters, households tended to have floors made with wood, and household density was lower. Treatment-seeking approaches differed significantly inside and outside the clusters. Government provided prevention/treatment programs were used much more frequently by people in the malaria cluster areas than among individuals outside these regions. However, we do not interpret this as indicating program failure (see below).

## Discussion

In the national level survey conducted in 2007, the study area had the highest malaria prevalence (36%) [5]. Within two years of intervention implementation the overall malaria prevalence was 11.5%. This was consistent with decreased malaria prevalence (2∼6%) during the low transmission season in Kuhalong, Bandarban – adjacent districts [7] where the prevalence had been 27% in 2007 [Building resources across community (BRAC) and International Centre for Diarrhoeal Disease Research, Bangladesh's (ICDDR,B) unpublished report]. In 2008, malaria morbidity and mortality in Bangladesh were recorded as 84,690 and 154, respectively [8]. By 2009 cases and confirmed deaths had decreased to 63,873 and 47 respectively [4]. Thus, malaria appears to have significantly decreased during the 2 years of the intervention program. However, prevalence still remained more than 10% and efficient control strategies are necessary to further reduce malaria risk. For effective malaria control in such areas, we need to identify high-risk groups and areas for more intensive targeting. The present study identified several socio-economic and environmental risk factors within the study area.

The prevalence of malaria was highest in younger 0–14 years old, males which is similar to that reported by the national malaria baseline survey [5] and other studies [9], [10], [11]. Presumably the age effect may be a consequence of fewer episodes and lower immunity. It may reflect differences in exposure risks. The significantly higher prevalence observed in these males compared females, however, differs from the previous nationwide survey, which showed no difference in malaria risk between sexes [5]. The higher infection rate among males in the study area may relate to behavioral differences among males and females, such as participation in agricultural and forest work activities [12]. This could also be explained by women having a preference to be well covered with clothing and generally sleeping earlier with their children under bed nets [12]. After adjusting for the covariates, ethnic group was not a significant predictor of malaria infection.

For our study area, the Global Fund project had a target LLIN distribution of 90% household coverage, and as reported previously [6], this target goal has been well achieved. LLIN coverage was high among our study population and we found no significant effect of the possession of LLIN on malaria infection. However, the number of bed nets in households was associated with malaria risk and households with ≥2 bed nets were at a reduced risk. This is consistent with other studies showing the impacts of additional bed nets in preventing infection in Bangladesh, Vietnam, and Somalia [5], [13], [14]. Thus, efforts should be focused on increasing the supply of LLINs to at least 2 per household in malaria-endemic areas of Bangladesh, especially in the CHT region. This is also in accordance with the objectives of the malaria control program in Bangladesh supported by Global Fund Round nine [15].

Forest coverage and high altitude also proved to be a significant risk factor for malaria infection in the study area. It might be due to environmental preference by the malaria vector, although the vector species have not been confirmed for this study area. In this region *Anopheles dirus*, *Anopheles minimus*, and a diverse fauna of other anopheline species have been reported as main malaria vectors [16], [17]. Several of these species prefer forest or forest fringe habitats [18], [19]. Univariate analysis further revealed that lower household density was a significant risk factor for malaria infection. This pattern is similar to a study from Ghana that reported higher malaria risk in smaller villages and in outer areas of each village [20]. However, for the multivariate model, the effect of household density was reversed and definitive conclusions could not easily be made. Strong correlations were observed between the forest coverage and house density, and may account for the results in the multivariate model.

Spatial analysis revealed that locations at increased risk of malaria were not uniformly distributed at the sub-district level. Three large clusters of high malaria risk were identified in the eastern part of the study area, which is mostly elevated and more forested. In addition, people living within the high-risk clusters were characterized by several socioeconomic risk factors.

Certain ethnic groups identified as at higher risk in the regression analysis such as Tipura and Tonchonga were exclusively limited to the high-risk clusters. Other social conditions such as housing structure (wood flooring) and lower household density were more frequent within the high-risk clusters. These findings suggest that any initiatives to control malaria in the near future could be more efficient by targeting specific areas where high-risk groups in terms of multiple risk factors are clustered.

The use of malaria treatment supported by the government or BRAC was higher within the high-risk clusters. This most likely reflects the recent government efforts to provide care to populations that previously received any interventions. These more remote areas remain regions where the drug venders, which are the alternative treatment measure, are not abundant. Because the usage of the appropriate treatment measure is already high (85.5% of households) within high-risk cluster, further improvements might be challenging.

There are several limitations with this study. First, as a cross-sectional survey conducted during the rainy season, when peak malaria transmission was expected, different results may have been obtained if a different time-frame had been chosen. However, we expect that this should give a conservative estimate of the impact of the current national control program as transmission should be near its peak. Additionally, the questionnaire used in this study evaluated only a subset of potential socioeconomic risk factors [21] as well as other potential environmental risk factors. For example, malaria in this region appears associated with the dynamics of precipitation patterns reflected in vegetation growth [22]. The absence of dynamical environmental factors such as hydrology-driven ecological factors directly affects vector population dynamics. Understanding these local topographic effects may allow for better prediction of regions at high risk of malaria within the forest-covered highlands on small spatial scales.

## Methods

### Study area

The study was conducted in the Rajasthali sub district of the Rangamati district, one of the 3 districts in the CHT in the southeastern region of Bangladesh. This sub district was chosen as it had the highest malaria prevalence (36%) in the survey conducted in 2007. Rajasthali contains a population of 24,097 people (50.3% male), living in 5,322 households. There are 6 ethnic communities residing in Rajasthali — Marma, Tripura, Tonchonga, Khiang, Chakma and Bengali. Marma is the largest ethnic group, and Bengali (the Bangladesh majority) is the second largest group in this area [6]. All households (n = 5322) in Rajasthali were georeferenced using a single hand-held GPS receiver (eTrex Venture, Garmin) from January until April 2009 [6].

### Ethical considerations

A consent form was developed and was approved by ICDDR, B research review committee and ethical review committee. Consent form was read in front of study participant. If he/she was under 18 years, consent forms was made clear to his/her legal guardian. It was also made clear in consent form about purpose of study, advantages and disadvantages. Participation in the study was completely voluntary. A signature was taken in consent form before participating in the study.

All malaria positives were referred for treatment to nearest BRAC health workers (Implementing malaria control program in Bangladesh) or government hospital. Malaria infected people were treated with artemether-lumefantrine (AL)(Coartem®).

### Sampling, malaria diagnosis and socioeconomic survey

In July 2009, a survey on malaria prevalence was conducted using a Rapid Diagnostic Test (RDT) kit. Due to logistical constraints 1,400 households were sampled from 5,322 (26.3%) households in Rajasthali. A 2-stage cluster sampling technique was used. In each village, the survey team selected every third household encountered as they moved from the periphery of the village following a designated path using a “spin the bottle” methodology [21]. From each selected household, we randomly selected an individual from a complete member's list using a lottery system. When selected member was not present in the household, we then moved to the next household and repeated the lottery approach. If any household denied participating, study team collected sample from the next or previous household.

A finger-prick blood sample was taken from each individual and tested using FalciVax (Zephyr Biomedicals) to detect *P. falciparum* and *Plasmodium vivax*-specific antigens. This diagnostic kit is reported to have a high correlation with microscopic examination and has been recommended to malaria control programs by the World Health Organization [23].

A questionnaire survey was conducted at each participating household to collect information on individual demographics, household heads education and occupation, the total number of bed nets owned by the household, the household ownership of LLINs, and the treatment of ITN or ordinary bed nets within the last 6 months, number of family members sleeping under a bed net, type of housing structure (such as floors) and treatment measures used against malaria [24]. LLIN ownership and bed net treatment was merged into a single variable as ‘treated bed net or LLIN ownership’ and analyzed. The questionnaire survey was conducted during a face-to-face interview with the head of each household. In the absence of a household head, the interview was conducted with any other adult in the household. In cases where inconsistencies were noted, interviewers were accompanied by the field supervisor until quality standards were met. Local interviewers from each ethnic group were hired to facilitate completion of the questionnaire survey for the various groups.

### Environmental variables

#### Land cover

**Figure 2:**
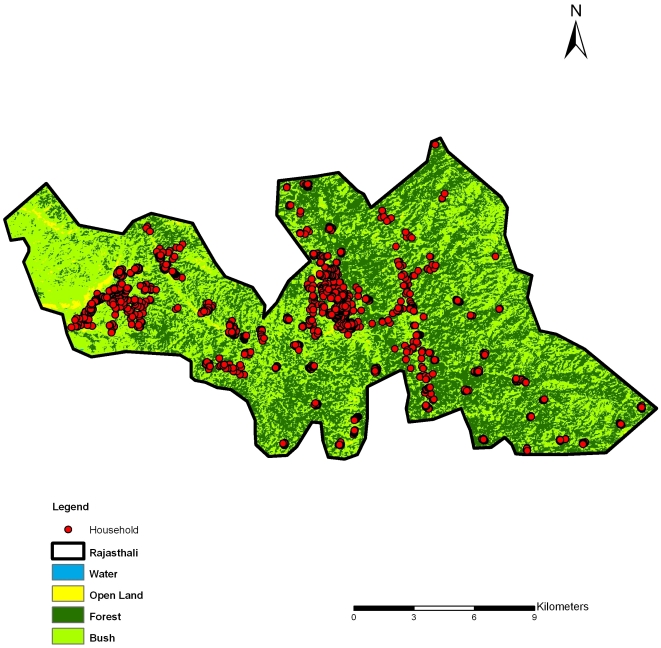
Land cover classification in Rajasthali.

A Landsat 5 Thematic Mapper image taken on January 23rd, 2010 (Path 136, Row 44) was obtained from USGS Global Visualization Viewer [25] and used for land-cover classification. Ground-truth sites and known land-covers were identified from high-resolution Google Earth images, and Regions of Interest were defined in the following 6 categories: deep water, shallow water, brown open land, bright open land, forest, and grassland/bush. The study area was classified into 6 categories based on supervised classification using the maximum likelihood method for PG-Steamer (Pixoneer Geomatics Inc., Tae-jon). As an environmental variable, the proportion of forested area (Figure 2) within 2 km from each of 1400 sampled households was calculated using Arc GIS 9.3 (ESRI, Redlands, CA) with the Hawth's Tool extension [26]. The buffer size was set to 2 km because the flight range of the malaria vector mosquitoes could be approximately considered at this scale [27]. The proportion of forested area was categorized into tertiles for the analysis.

#### Altitude

A Shuttle Rader Topographic Mission Digital Elevation Model (SRTM DEM) of 3 arc-second (approximately 90 m) resolution was used to estimate the altitude of each sampled household. After conversion to a Geo TIFF file using PG-Steamer, the altitude data for 1400 houses were extracted using Arc GIS 9.3 with a Spatial Analyst extension. The altitude was categorized into 3 classes for statistical analysis: ≤50 m, 51–100 m, and >100 m.

#### Local house density

As another environmental variable, we quantified local house density for 1400 sampled households using GPS location of census houses. With GPS coordinates projected in the Universal Transverse Mercator (UTM 46N) system, the distance from each sampled house to all other households identified in census was calculated and the number of houses within a buffer area was counted using Excel 2007 (Microsoft). For the same reason as above, the buffer size was set to 2 km. The number of houses was categorized into following classes for statistical analysis: 1–200, 201–500, 501–1000, and >1000.

### Data analysis

#### Spatial analysis

SaTScan (v. 8.2.1) was used to detect spatial clusters (adjusted for more likely clusters) of malaria (settings: spatial analysis; Bernoulli probability model; latitude/longitude coordinates; no geographical overlap; scanning for clusters with high rates). Spatial clusters were determined by calculating the maximum likelihood ratio. Standardized prevalence ratios were estimated by dividing the number of observed cases by the number of expected cases in each cluster. Simulated p values were obtained using Monte Carlo methods with 999 replications [28].

#### Risk factor analysis

Univariate analyses were performed using logistic regression to determine the association between malaria prevalence and potential risk factors. All variables with a p value less than 0.05 for likelihood ratio test in univariate models were entered into multivariate logistic regression models. We then compared the risk factors of cases in a spatial cluster identified by the SaTScan to those of cases outside of a cluster using univariate and multivariate logistic regression analysis. This analysis provides further insights into the underlying factors that characterize malaria risk specifically in high-risk areas. Statistical analysis was performed with STATA 11 (Stata Corp. 2003, College Station, TX).
